# Correlation of body mass index levels with menarche in adolescent girls in Shaanxi, China: a cross sectional study

**DOI:** 10.1186/s12905-016-0340-4

**Published:** 2016-09-06

**Authors:** Zhenjie Wang, Shaonong Dang, Yuan Xing, Qiang Li, Hong Yan

**Affiliations:** 1 Institute of Population Research/WHO Collaborating Center on Reproductive Health and Population Science, Peking University, No.5 Yiheyuan Road, Haidian District Beijing, 100871 People’s Republic of China; 2 Department of Epidemiology and Health Statistics, School of Medicine, Xi’an Jiaotong University, Xi’an, 710061 People’s Republic of China; 3 Xi’an center for disease control and prevention, Xi’an, 710061 People’s Republic of China

**Keywords:** Adolescent girls, Body mass index, Menarche, Western China

## Abstract

**Background:**

Menarche is a milestone for adolescent girls. The timing of menarche is influenced by genetics, social status and nutritional status (e.g., height, weight and body mass index [BMI]) and impacts future health (e.g., obesity and breast cancer). There have been many studies on trends in age at menarche among adolescent girls in China, but few have investigated associations between growth status and the timing of menarche. This study examined the association between age at menarche and growth status among adolescent girls in Western China.

**Methods:**

The participants in this cross sectional study came from three geographical regions of Shaanxi Province. A total of 533 adolescent girls from urban and rural areas were randomly selected. Trained investigators administered a standard questionnaire to each participant during a face-to-face interview and carried out anthropometric measurements.

**Results:**

The average age at menarche was 13.3 years. There were statistically significant differences in BMI z-scores between pre-menarcheal and post-menarcheal girls of the same age and these differences were related to socioeconomic factors. Girls who had reached menarche, in particular those aged 13–14 years, were significantly taller (*P* < 0.01) and had higher BMI (*P* < 0.01) than girls in the same age group who had not reached menarche.

**Conclusions:**

BMI is associated with the timing of menarche but socioeconomic factors are also important.

## Background

Currently, the age at menarche averages between 12 and 13 years worldwide [1]. Over the last century it has decreased by 2 to 3 months per decade in the United States and Europe [1]. In Asian countries, there is also evidence of a secular trend toward earlier menarche [1–7]. Menarcheal age is influenced by diverse variables such as genetics, geographic location, climate, psychological factors, socioeconomic status, body weight and height, nutrition, body fat and exercise, as well as the presence of chronic diseases [8–11].

In China, a particularly steep decline in the age at menarche in some areas has been attributed to rapid improvements in nutritional status, with children and adolescents steadily gaining more weight and height over the past 30 years [12, 13]. For example, in Beijing the mean menarcheal age was 14.2 years in a 1962 cohort and 12.7 years in a 1985 cohort, a difference of 1.5 years or a decline of 8 months per decade [14, 15]. Age at menarche has been reported to be associated with growth status as reflected by height, weight and body mass index (BMI) [16]. However, Stark et al. [17] suggested that the role of nutrition in determining age at menarche is relatively unimportant in affluent individuals. Other epidemiologic studies indicate that adolescent girls who have reached menarche gain more weight and height and therefore have higher BMIs than girls of the same age who have not started menstruating [17]. Consequently, adolescent girls may have a BMI in the overweight range because of early onset of puberty rather than simply because of long-term positive energy balance and excess fat [18]. Age at menarche may also represent an important clinical and public health indicator of susceptibility to obesity in adulthood, with its attendant morbidity [19].

Age at menarche in several regions of China has been previously reported [2–7]. However, most studies have focused on populations from relatively developed areas, such as the coastal areas of China, and on long-term trends in the age at menarche. Very few studies [2] have addressed the associations between height, weight, BMI and menarche among adolescent girls in less developed areas, such as the northwest and southwest of China. The aim of this study is to investigate the relationship between age at menarche and socioeconomic factors in a population from Shaanxi Province and to examine the relationship between menarcheal age and growth status indicators, such as height, weight and BMI.

## Methods

### Setting and survey design

Shaanxi Province comprises three distinct geographic regions: the northern plateau (Loess Plateau), the southern region (south of the Qin Mountains) and the Guanzhong plain (between the Loess Plateau and the Qin Mountains and including the lower Wei River valley). There are considerable differences among these three regions in terms of economic development. In 2006 there were approximately 18 million female inhabitants in Shaanxi Province [20]. The current cross sectional survey was part of a survey carried out across Shaanxi Province between July and August 2007 to determine primary health and nutritional status indicators among adolescent girls and adult females aged 20–49 years and to compare these indicators among the three geographic regions. The study was approved by the Ethics Review Committee at Xi’an Jiaotong University School of Medicine. All participants provided written informed consent. For girls aged younger than 16 years, consent was obtained from parents or guardians. The Department of Epidemiology and Health Statistics, School of Medicine, Xi’an Jiaotong University gave permission for the use of the data in the current report.

### Sampling

Within Shaanxi Province, where appropriate, sampling strata were defined based on local geographic characteristics (three geographic regions) to allow for anticipated regional variability. Within each stratum, a four-stage sampling strategy was followed involving four natural administrative units (i.e., county, township, village and community) and sampling was conducted with probability proportional to cluster size. Eighty counties in rural areas and 20 counties in urban areas were randomly sampled based on population proportion. Next, one township was randomly selected from each sampled county in the urban areas. One or two villages were also selected from each sampled township in rural areas. Based on the list of inhabitants in selected villages or communities, 20 adult females aged 20–49 and 20 adolescent girls aged younger than 20 years were randomly sampled. The primary aims of this survey were to evaluate the status of health and nutrition among the study population and to investigate the association between socioeconomic factors and health status among adult females and girls in Shaanxi province. The calculated sample size was 1 200 participants, including 600 adult females aged between 20 and 49 years and 600 girls aged younger than 20 years. A total of 642 adult females (415 from rural areas and 217 from urban areas) and 561 girls (396 from rural areas and 165 from urban areas) completed the survey. Data from 533 adolescent girls were used in the current analysis after the exclusion of missing values.

### Data collection and measurement

Face-to-face interviews and a standard questionnaire were used to collect information on household background, health status and reproductive history. Participants were asked whether they had reached menarche. Those who answered affirmatively were then asked to recall the year of menarche. To calculate the average age of the onset of menstruation, probity models were fitted to the proportion of girls at each age who had reached menarche. This approach, sometimes referred to as the status quo method, is mainly used in cross sectional studies of children and adolescents [21–23]. The status quo method is employed to estimate the mean age at menarche by studying all data concerning girls from the analysed group, whereas the retrospective method can only be used for girls who have already reached menarche [21]. The calculation was carried out as follows: ages were recorded and calculated as decimals (e.g., 8.00–8.99 years, 9.00–9.99 years). The proportion of girls who had reached menarche was transformed into *y*, a normal equivalent deviate

$$ \mathrm{P}={\displaystyle \underset{-\infty }{\overset{y}{\int }}}f\left(\theta \right)d\theta,\ where\ y=a+bx $$

is the age in months and *a* and *b* are the parameters to be estimated from the data.

The participants were asked to remove any type of hairstyles that increased height and to remove all heavy clothes, shoes and accessories. Trained staff weighed the participants on a calibrated electronic scale (Tanita HD-305, Tanita Trading Co., Ltd., Shanghai, China) and recorded the value to the nearest 0.1 kg. A height measuring tape (LD-SG01, Ningbo Land Corporation, Zhejiang, China) was used to measure standing height. Subjects stood erect with their shoulders level, hands at their sides and thighs and heels comfortably together. The subjects also kept their upper back, buttocks and heels in contact with the wall and their head aligned in the vertical plane. Height was recorded to the nearest 0.1 cm. A single trained staff member in each field team performed all anthropometric measurements. BMI was calculated as weight in kg/height in m^2^. The Centres for Disease Control and Prevention (CDC) BMI-for-age percentile cutoff points for girls aged 2–20 years were used to classify BMI into the following percentile categories: ≤5th, 5th to ≤10th, 10th to ≤25th, 25th to ≤75th, 75th to ≤85th and 85th percentile [24, 25]. BMI z-scores were calculated using the CDC’s software package Epi Info™ version 3.5.1 (Nutrition) based on BMI-for-age reference standards for girls aged 0–20 years.

### Quality control

Prior to data collection and survey interviews, a training meeting was held in Xi’an for all team members. Three field teams were established according to the geographic distribution of sample sites. Each team consisted of four members responsible for collecting blood, isolating serum, interviewing subjects and collecting anthropometric data. Staff from the county health bureau or maternal and child health care station coordinated the activities of each team at each sample site. Doctors from village clinics or township hospitals were also involved in fieldwork to ensure preparation in the event of a medical emergency. To reduce recall biases for age at menarche, quality control methods were adopted to assist with recall, such as prompting subjects to remember special events (e.g., the Chinese festival).

After each interview, investigators checked all information collected through the questionnaire. If logic errors were found in the obtained information, interviews were repeated. Key members of each team also crosschecked all questionnaires at each site, with repeat interviews carried out as needed. Finally, staff of the School of Medicine at Xi’an Jiaotong University checked all questionnaires.

All measuring devices were calibrated, standard procedures were followed, a program of logical control and check was established and duplicate data entry was used.

### Statistical analysis

The study database was established using EpiData version 3.1 (EpiData Association, Odense, Denmark). Mean ± standard deviation (SD) was used for the description of continuous variables and percentages were used for categorical data. Age at menarche was calculated by probity regression analysis. The chi-square test and Fisher’s exact test were used to examine differences between ratios of geographic characteristics, BMI percentiles and distribution of age at menarche. The associations between the onset of menarche and socioeconomic factors were examined by generalized linear model. The two-sample *t*-test was used to examine differences between pre-menarcheal and post-menarcheal growth status. All tests were two-tailed. A *P* value of <0.05 was considered statistically significant. SAS version 9.2 (SAS Institute Inc., Cary, NC, USA) was used for all analyses.

## Results

**Fig. 1:**
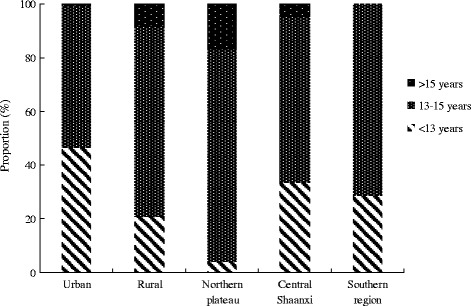
Distribution of age groups of menarche based on rural and urban and geography site†

**Fig. 2:**
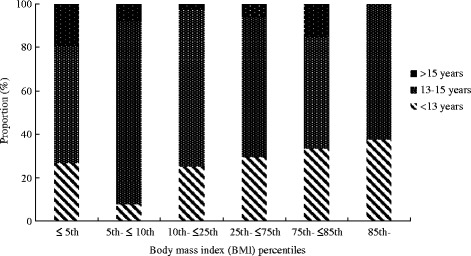
Distribution of age groups of menarche based on body mass index (BMI) percentiles†

The characteristics and BMI distribution of adolescent girls are presented in Table 1. Of the 533 participants, 83.5 % (*n* = 445) reported that they had reached menarche. The average age at menarche was 13.3 years. The distribution of age at menarche according to geographic characteristics and BMI percentile is shown in Figs. 1 and 2, respectively. There were significant differences among menarcheal age groups related to urban-rural distribution, geographical area and BMI percentile. Most girls were junior high school students and living in rural areas. The most common source of family income was from farming or farming plus other sources. Urban girls reported an earlier age of menarche than their rural peers (12.7 years vs. 13.5 years, *P* < 0.001, data not shown). BMI z-scores for adolescent girls according to geographic characteristics and menarcheal status are presented in Table 2. There were statistically significant differences in BMI z-scores between pre-menarcheal and post-menarcheal girls according to education, family size, source of income, rural vs. urban environment and geographic location. BMI z-scores were lower in pre-menarcheal girls than in post-menarcheal girls.

**Table 1:** Characteristics of adolescent girls by selected variables

Variables	Outcome	
Age (years), Mean ± SD	15.0 ± 1.22	
Education (years), Mean ± SD	8.3 ± 1.55	
Age at menarche, Mean ± SD	13.3 ± 1.33	
Family size(persons), Mean ± SD	4.3 ± 1.09	
Body mass index (BMI) percentiles,	*n*	%
≤ 5th	286	53.7 %
5th- ≤ 10th	53	9.9 %
10th- ≤25th	42	7.9 %
25th- ≤75th	104	19.5 %
75th- ≤85th	28	5.3 %
85th-	20	3.8 %
Rural and urban		
Urban	144	27 %
Rural	389	73 %
Geography site		
Northern plateau	92	17 %
Central Shaanxi	383	72 %
Southern region	58	11 %
Source of income		
Farming only	153	28.7 %
Farming plus others	122	23.9 %
Others	258	48.4 %

**Table 2:** Comparison in body mass index (BMI) Z-scores between menstruating (post-) and non-menstruating (pre-) adolescent girls by the characteristics of adolescent girls

	BMI Z-scores, Mean ± SD		*P* ^a^
	Pre-	Post-	
Education			
< 6	−1.10 ± 0.78	−0.57 ± 0.77	0.19
6–9	−1.15 ± 1.01	−0.30 ± 0.81	<0.01
> 9		−0.33 ± 0.88^b^	
Family size			
< 3		−0.05 ± 0.69^b^	
3–5	−1.18 ± 1.05	−0.29 ± 0.81	<0.01
> 5	−1.12 ± 0.91	−0.58 ± 0.84	0.03
Source of income			
Farming only	−1.13 ± 0.83	−0.37 ± 0.79	<0.01
Farming plus others	−1.5 ± 0.78	−0.43 ± 0.86	<0.01
Others	−0.90 ± 1.26	−0.23 ± 0.82	0.01
Rural and urban			
Urban	−1.08 ± 1.16	−0.19 ± 0.83	<0.01
Rural	−1.20 ± 0.97	−0.36 ± 0.81	<0.01
Geography site			
Northern plateau	−1.22 ± 1.23	−0.40 ± 0.91	<0.01
Central Shaanxi	−1.15 ± 1.01	−0.30 ± 0.81	<0.01
Southern region	−1.21 ± 0.77	−0.27 ± 0.71	<0.01

^a^Two-sample *t*-test was used to examine differences between pre- and post- menstruating

^b^No subjects were observed in non-menstruating (pre-) category

The generalized linear model (*r*^2^ = 0.30, *P* < 0.01) did not identify any statistically significant associations between the onset of menarche and the following: urban/rural environment, geographic location, education, family size or source of income. However, there were significant associations between the onset of menarche, age (*β* = −0.12, *P* < 0.01) and BMI (*β* = −0.03, *P* < 0.01). After the adjustment for all other variables, low BMI was associated with delayed onset of menarche among adolescent girls.

**Fig. 3:**
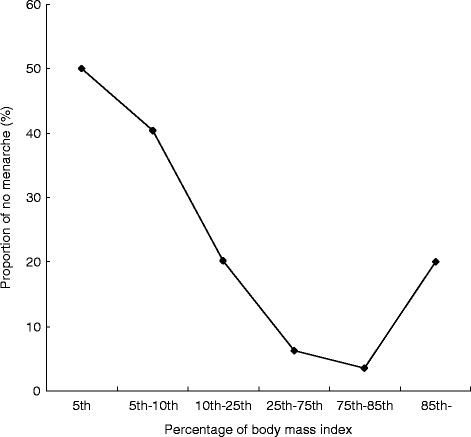
Association between percentage of body mass index (BMI) and proportion of no menarche

**Fig. 4:**
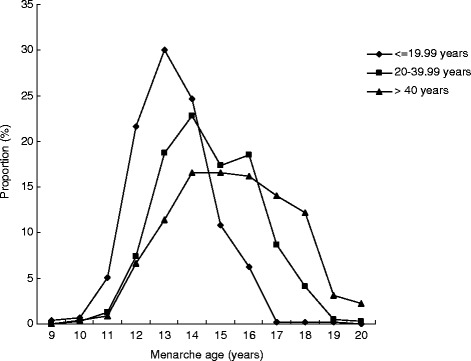
Distribution of age at menarche in different age groups

Table 3 compares anthropometric data according to menarcheal status. The mean values for height, weight, BMI and BMI z-scores were higher among post-menarcheal girls compared with their pre-menarcheal peers. Mean height and weight were significantly higher for menstruating girls in nearly all age groups, and there were significant differences in BMI between girls aged 13–14 years who had reached menarche and those who had not. The proportion of pre-menarcheal girls decreased up to the 85th percentile for BMI, and then increased from that point (Fig. 3). The distribution of menarcheal age by birth cohort is presented in Fig. 4. The median menarcheal age fell in conjunction with age group. Menarche occurred at approximately 13 years in those born after 1988, 14 years in girls born between 1968 and 1988 and 15 years in those born before 1968.

**Table 3:** Growth status of menstruating (post-) and non-menstruating (pre-) adolescent girls

Age	*n*		Height (cm), Mean ± SD		Weight (kg), Mean ± SD		BMI (kg/m^2^), Mean ± SD		BMI Z-scores, Mean ± SD	
	Pre-	Post-	Pre-	Post-	Pre-	Post-	Pre-	Post-	Pre-	Post-
<12	5	0	141.8 ± 4.14		30.4 ± 2.85		15.1 ± 0.60		−1.35 ± 0.40	
12–12.99	9	6	147.4 ± 6.31	154.1 ± 4.02^*^	35.3 ± 4.25	41.2 ± 3.51^*^	16.2 ± 1.70	17.3 ± 1.00	−1.12 ± 0.84	−0.51 ± 0.43
13–13.99	38	52	150.2 ± 6.90	155.1 ± 4.92^**^	38.4 ± 9.24	45.1 ± 5.84^**^	17.0 ± 3.69	18.7 ± 2.21^*^	−1.15 ± 1.06	−0.34 ± 0.84^**^
14–14.99	29	132	150.8 ± 7.73	155.9 ± 5.76^**^	38.4 ± 8.91	47.0 ± 6.46^**^	16.8 ± 2.85	19.3 ± 2.27^*^	−1.29 ± 1.10	−0.23 ± 0.77^**^
15–15.99	7	138	151.5 ± 7.35	157.9 ± 5.26^**^	43.0 ± 6.20	49.2 ± 6.51^*^	18.7 ± 1.88	19.7 ± 2.21	−0.68 ± 1.03	−0.29 ± 0.84
16–16.99	0	85		157.7 ± 5.47		49.4 ± 5.58		19.9 ± 2.11		−0.39 ± 0.80
17–17.99	0	18		155.3 ± 4.09		49.6 ± 5.87		20.6 ± 2.45		−0.30 ± 0.92
18–19.99	0	6		158.4 ± 2.96		51.0 ± 7.54		20.3 ± 3.24		−0.67 ± 1.40

^*^P < 0.05, ^**^P < 0.01

## Discussion

The current study showed that the age of menarche has been decreasing gradually in Western China across different birth cohorts. Girls who reached menarche were taller, heavier and had higher BMIs compared with their premenstrual peers.

Previous studies have reported that the average age at menarche for healthy adolescent girls is approximately 13.3 years in Shanghai, 12.9 in Guangzhou, 12.7 in Tianjin, 12.9 in Harbin and 13.2 in Qingdao [3–7]. In the current study, the average age at menarche was the same as that reported in the study conducted in Shanghai, but higher than that reported for other regions. Because the average age at menarche was generally decreasing, the difference among survey years could have contributed to the different results. However, the age at menarche for all birth groups showed a decreasing trend, consistent with existing data for Chinese populations [2–7].

Economic conditions may largely explain the earlier onset of menarche among urban girls compared with their rural counterparts. Our results add to evidence that economic conditions and standard of living have a major influence on menarche among adolescent girls in Western China. On-going improvement in living standards in both urban and rural areas may lower the age of menarche [26, 27]. Despite China’s recent economic growth, the gap between urban and rural incomes is still large and increasing. Although costs of living are higher in urban areas, residents have better nutritional status and a higher standard of health care than their rural counterparts. Observational studies suggest that living in an urban vs. rural environment in China has a considerable effect on the age of onset of menarche [26, 27]. The current study also found that urban girls reported an earlier age of menarche than their rural peers. Moreover, results of studies conducted in other undeveloped countries indicate that girls who have reached menarche are significantly taller, heavier and have higher BMIs than pre-menstrual girls in the same age group [28]. Gain in body fat could be one of the key factors involved, possibly through secretion of the fat-derived protein leptin, which stimulates the hypothalamus to increase secretion of gonadotropin-releasing hormone [29]. Our findings also suggest that adolescent girls aged 13–14 years who have reached menarche are significantly taller, heavier and have higher BMIs than girls in the same age group who have not started menstruating.

Some of the differences in the distribution of age at menarche could be related to environmental variables that were beyond the scope of this cross sectional study. Early age at menarche could cause increasing body weight and higher BMI that can persist in the post-menarcheal period. The current study suggests the latter, and indicates a declining trend in the age of menarche in western Chinese adolescents. Furthermore, there was a delayed trend in menarche among adolescents in the high BMI percentiles. This might be another effect modification of the association of BMI with menarche associated with socioeconomic status.

This study was limited by the small sample size and cross sectional design. These limitations reduce the generalizability of the results and do not provide evidence of causality. Furthermore, because of the small sample size, it is also difficult to examine the effect of modification of age at menarche by socioeconomic status. In addition, the adolescent growth spurt was not considered in the current study. Given these limitations, the current results should be interpreted with caution and further investigation is warranted to expand on the findings. However, the study also has strengths such as the random selection of investigation areas and participants. The findings provide useful information on the age of menarche in adolescent girls in western China.

## Conclusions

The timing of menarche is influenced by many factors, and a trend towards earlier onset of menarche has been observed in Western China. This study investigated associations between height, weight, BMI and menarche among adolescent girls, and found that BMI is an important indicator of the timing of menarche.
